# F159. NEUROMAGNETIC 40 HZ AUDITORY STEADY STATE RESPONSES AND AUDITORY CORTICAL GABA AND GLX IN CLINICAL HIGH RISK AND FIRST EPISODE OF PSYCHOSIS INDIVIDUALS

**DOI:** 10.1093/schbul/sby017.690

**Published:** 2018-04-01

**Authors:** Hanna Thuné, Tineke Grent-’t-Jong, Emmi Mikanmaa, Marc Recasens, Frances Crabbe, Jozien Goense, Andrew Gumley, Matthias Schwannauer, Stephen Lawrie, Ruchika Gajwani, Joachim Gross, Peter Uhlhaas

**Affiliations:** 1 University of Glasgow; 2 University of Edinburgh; 3 Royal Edinburgh Hospital

## Abstract

**Background:**

Robust impairments in the power and phase of 40 Hz auditory steady state responses (ASSR) have been reported in chronic schizophrenia patients. This could reflect changes in the balance between inhibitory GABAergic and excitatory glutamatergic neurotransmission in auditory cortex. However, the direct link between the ASSR and alterations in these neurotransmitter systems has not been systematically explored. Furthermore, it remains unclear whether 40 Hz ASSR impairments are present in early and at-risk stages of psychosis. The current study aims to explore the 40 Hz ASSR in first-episode of psychosis patients and individuals at clinical high risk (CHR) of psychosis, and the possible relationship of deficits in gamma-band entrainment to a dysfunctional excitation inhibition balance, as reflected by alterations in cortical GABA and glutamate.

**Methods:**

Data from 80 CHR, 11 FEP and 40 age-matched healthy control participants were collected as part of the MRC-funded Youth Mental Health Risk and Resilience study. MEG data were recorded on a 4D Neuroimaging Magnes 3600 Whole Head 248 Channel system, while participants were passively presented with a series of 1000 Hz carrier tones amplitude modulated at 40 Hz. Data were analysed at sensor and source-level in the frequency-domain for spectral power and intertrial phase-coherence (ITPC). For source-reconstruction, an eLoreta source-analysis algorithms was employed. Auditory regions of interest (ROIs) were defined using 98 nodes defined from the AAL-atlas. Levels of right auditory GABA and Glx (glutamate + glutamine) were measured using 1H-MRS at 3T and 2*2*2 cm voxels and were estimated relative to water. GABA levels were further corrected for grey and white matter and cerebrospinal fluid levels within the voxel. Finally, 40 Hz ASSR power in right auditory cortex was explored in relation to neurotransmitter levels in the same region.

**Results:**

Across groups, the ASSR stimulus activated temporal regions, including bilateral heschl’s gyrus and superior temporal cortex. A significant effect of hemisphere was found, reflecting higher 40 Hz ASSR power in the right hemisphere across groups. CHR and FEP participants showed attenuated 40 Hz ASSR power and ITPC compared to healthy control participants in right temporal regions, but an increase in spectral power in the left hemisphere. A moderate positive correlation was found between right auditory GABA and 40 Hz ASSR power in the right superior temporal gyrus in CHR, but not in controls.

**Discussion:**

These results provide a link between MRS measures of GABA and 40 Hz ASSR power impairments in CHR individuals. Furthermore, these preliminary findings indicate that slight alterations in the 40 Hz ASSR are present in FEP patients and may arise already in the CHR stage, prior to the onset of psychosis.

